# Anti-ulcerative colitis effects of chemically characterized extracts from C*alliandra haematocephala* in acetic acid-induced ulcerative colitis

**DOI:** 10.3389/fchem.2024.1291230

**Published:** 2024-02-27

**Authors:** Inaam Ur Rehman, Mohammad Saleem, Syed Atif Raza, Saher Bashir, Taha Muhammad, Shahzad Asghar, Muhammad Usman Qamar, Tawaf Ali Shah, Yousef A. Bin Jardan, Amare Bitew Mekonnen, Mohammed Bourhia

**Affiliations:** ^1^ Punjab University College of Pharmacy, University of the Punjab, Lahore, Pakistan; ^2^ Department of Chemistry, Faculty of Sciences, University of the Punjab, Lahore, Pakistan; ^3^ Shalamar Medical and Dental College, Lahore, Pakistan; ^4^ Department of Pharmacy, University of South Asia, Lahore, Pakistan; ^5^ Institute of Microbiology, Faculty of Life Sciences, Government College University Faisalabad, Faisalabad, Pakistan; ^6^ Division of Infectious Diseases, Department of Medicine, Geneva University Hospitals, Geneva, Switzerland; ^7^ Department of Microbiology and Molecular Medicine, University of Geneva, Geneva, Switzerland; ^8^ College of Agriculture Engineering and Food Science, Shandong University of Technology, Zibo, China; ^9^ Department of Pharmaceutics, College of Pharmacy, King Saud University, Riyadh, Saudi Arabia; ^10^ Department of Biology, Bahir Dar University, Bahir Dar, Ethiopia; ^11^ Laboratory of Biotechnology and Natural Resources Valorization, Faculty of Sciences of Agadir, Ibnou Zohr University, Agadir, Morocco

**Keywords:** ulcerative colitis, *Calliandra haematocephala*, HPLC, oxidative stress, pro-inflammatory markers, anti-inflammatory cytokines

## Abstract

**Background:** Ulcerative colitis is a chronic immune-mediated inflammatory bowel disease that involves inflammation and ulcers of the colon and rectum. To date, no definite cure for this disease is available.

**Objective:** The objective of the current study was to assess the effect of *Calliandra haematocephala* on inflammatory mediators and oxidative stress markers for the exploration of its anti-ulcerative colitis activity in rat models of acetic acid-induced ulcerative colitis.

**Methods:** Methanolic and n-hexane extracts of areal parts of the plant were prepared by cold extraction method. Phytochemical analysis of both extracts was performed by qualitative analysis, quantitative methods, and high-performance liquid chromatography (HPLC). Prednisone at 2 mg/kg dose and plant extracts at 250, 500, and 750 mg/kg doses were given to Wistar rats for 11 days, which were given acetic acid on 8th day through the trans-rectal route for the induction of ulcerative colitis. A comparison of treatment groups was done with a normal control group and a colitis control group. To evaluate the anti-ulcerative colitis activity of *Calliandra haematocephala*, different parameters such as colon macroscopic damage, ulcer index, oxidative stress markers, histopathological examination, and mRNA expression of pro and anti-inflammatory mediators were evaluated. mRNA expression analysis was carried out by reverse transcription quantitative real-time polymerase chain reaction (RT-qPCR).

**Results:** The phytochemical evaluation revealed polyphenols, flavonoids, tannins, alkaloids, and sterols in both extracts of the plant. Results of the present study exhibited that both extracts attenuated the large bowel inflammation and prevented colon ulceration at all tested doses. Macroscopic damage and ulcer scoreswere significantly decreased by both extracts. Malondialdehyde (MDA) levels and nitrite/nitrate concentrations in colon tissues were returned to normal levels while superoxide dismutase (SOD) activity was significantly improved by all doses. Histopathological examination exhibited that both extracts prevented the inflammatory changes, cellular infiltration, and colon thickening. Gene expression analysis by RT-qPCR revealed the downregulation of pro-inflammatory markers such as tumor necrosis factor-alpha (TNF-α) and cyclooxygenase-2 (COX-2) whereas the anti-inflammatory cytokines including Interleukin-4 (IL-4) and Interleukin-10 (IL-10) were found to be upregulated in treated rats.

**Conclusion:** It was concluded based on study outcomes that methanolic and n-hexane extracts of *Calliandra haematocephala* exhibited anti-ulcerative colitis activity through modulation of antioxidant defense mechanisms and the immune system. In this context, *C. haematocephala* can be considered as a potential therapeutic approach for cure of ulcerative colitis after bioassay-directed isolation of bioactive phytochemicals and clinical evaluation.

## 1 Introduction

Ulcerative colitis (UC) is a chronic bowel disorder resulting in disruption of the integrity of the colon as characterized by persistent colorectal inflammation and ulceration. No single collective etiology underlies the pathology of UC and instead, it has a multifactorial course involving environmental, hereditary, and immune-mediated causes (Shibrya et al., 2023). The incidence of UC in different regions of the world ranges from 0.6 to 24.3 per 100,000 individuals. Males and females are equally affected (Chashkova et al., 2023). Deregulation in pro and anti-inflammatory cytokine balance plays a vital role in the pathogenesis of UC (Badr et al., 2020). The key pro-inflammatory mediators in the pathogenesis of UC are tumor necrosis factor-alpha (TNF-α) and nuclear factor kappa B (NF-κB). TNF-α is released in response to harmful stimuli which in turn activates the NF-κB leading to the activation of inflammatory cascade via increased expression of inflammatory mediators (Salama et al., 2022). NF-κB signaling also upregulates the production of cyclooxygenase-2 (COX-2) which has a well-established pivotal role in the pathogenesis of UC (Zhao et al., 2023). Neutrophils and macrophages in the colon wall are activated by TNF-α (Jang et al., 2021). Oxidative stress in close communication with immune crisis also contributes to UC pathology due to an imbalance between antioxidant enzyme systems such as superoxide dismutase (SOD) and the number of oxidizing substances (da Fonseca et al., 2023). Activated macrophages and neutrophils are responsible for the generation of nitric oxide which in turn triggers lipid peroxidation and excess of malondialdehyde is produced. Inflammatory and structural changes in UC are associated with this oxidative and nitrosative stress (Balmus et al., 2016). In the context of this scenario, inflammation and oxidative stress are claimed to sketch UC (Bastaki et al., 2022). These underlying mechanisms result in pathological features of UC including macroscopic damage such as swelling, edema, erosion, and bleeding and microscopic alterations such as cellular infiltration, and necrosis of the colon ultimately resulting in the clinical course of the disease (Wu et al., 2022). Clinically, this disease is presented by abdominal pain, diarrhea, melena, weight loss and in complicated cases it can take transformation into carcinoma of colon (Elbastawisy et al., 2022).

Due to restricted comprehension of the precise etiology of this disease, treatment is critically undetermined, and primarily the management of inflammation is targeted in preference to other reasons. Aminosalicylates, immunosuppressants, glucocorticoids, and biological agents are the currently used four types of drug classes for the treatment of ulcerative colitis. However, devastating adverse effects, hormone dependence, drug resistance, and high economic costs are unsolved limitations associated with these available treatments (Liu et al., 2022). Furthermore, these medicines only induce and maintain remission, reduce the risk of complications, and improve some quality of life as far as administered (Gajendran et al., 2019). Moreover, these available treatments have low therapeutic effectiveness restricting the slowness of disease progression and even sometimes failing to produce any response (Shahid et al., 2022). Most importantly, there is no cure for ulcerative colitis to date which is a challenging fact concerning its treatment and it is the reason that life-long management of this disease is required (Lu et al., 2023). Additionally, available pharmacological therapies have single pharmacological targets in contrast to ulcerative colitis which has a multifactorial pathogenesis (Kang et al., 2022).

Downsides associated with available allopathic remedies necessitate the development of multi-oriented, efficacious, and safe therapies for ulcerative colitis. Herbal mode of therapy is gaining much attention worldwide as an alternative approach (Arunsi et al., 2022). Herbs have the potential to cure gastrointestinal issues including ulcerative colitis (Dubey et al., 2020). Plant-based medicines interact with multiple targets in pathophysiological pathways of ulcerative colitis to cope with the multifactorial nature of the disease (Yang et al., 2022). The herbal mode of treatment offers the least toxicity, favorable efficacy, and minimum economic cost (Yatoo et al., 2018).

*Calliandra haematocephala (c. haematocephala)*, commonly known as a red powder puff, belongs to *Mimosaceae* family. This plant is native to South America and is known for its bright red flowers and attractive foliage. *C. haematocephala* is a herbaceous plant that can grow up to a height of 1–3 m and has branches that are brown, cylindrical, and rough. Several pharmacological properties have been attributed to *C. haematocephala*, including anticonvulsant, anti-inflammatory, immunomodulatory, and gastroprotective activity. Its decoction has antioxidant activity and is used as a blood purifier (Punnagai et al., 2017). A phytochemical investigation of *C. haematocephala* revealed the presence of a range of compounds, including flavonoids, carbohydrates, alkaloids, saponins, phenolics, steroids, glycosides, and tannins, in plant leaves (Iftikhar et al., 2020). The plant’s butanolic extract has also been reported to have gastroprotective effects against acute gastric lesions (Antony, 2014). An effervescent granule formulation of *C. haematocephala* was developed in a previous work and the fondings were excellent in cntext of good flow properties of the formulation (Gupta et al., 2013).

**FIGURE 1:**
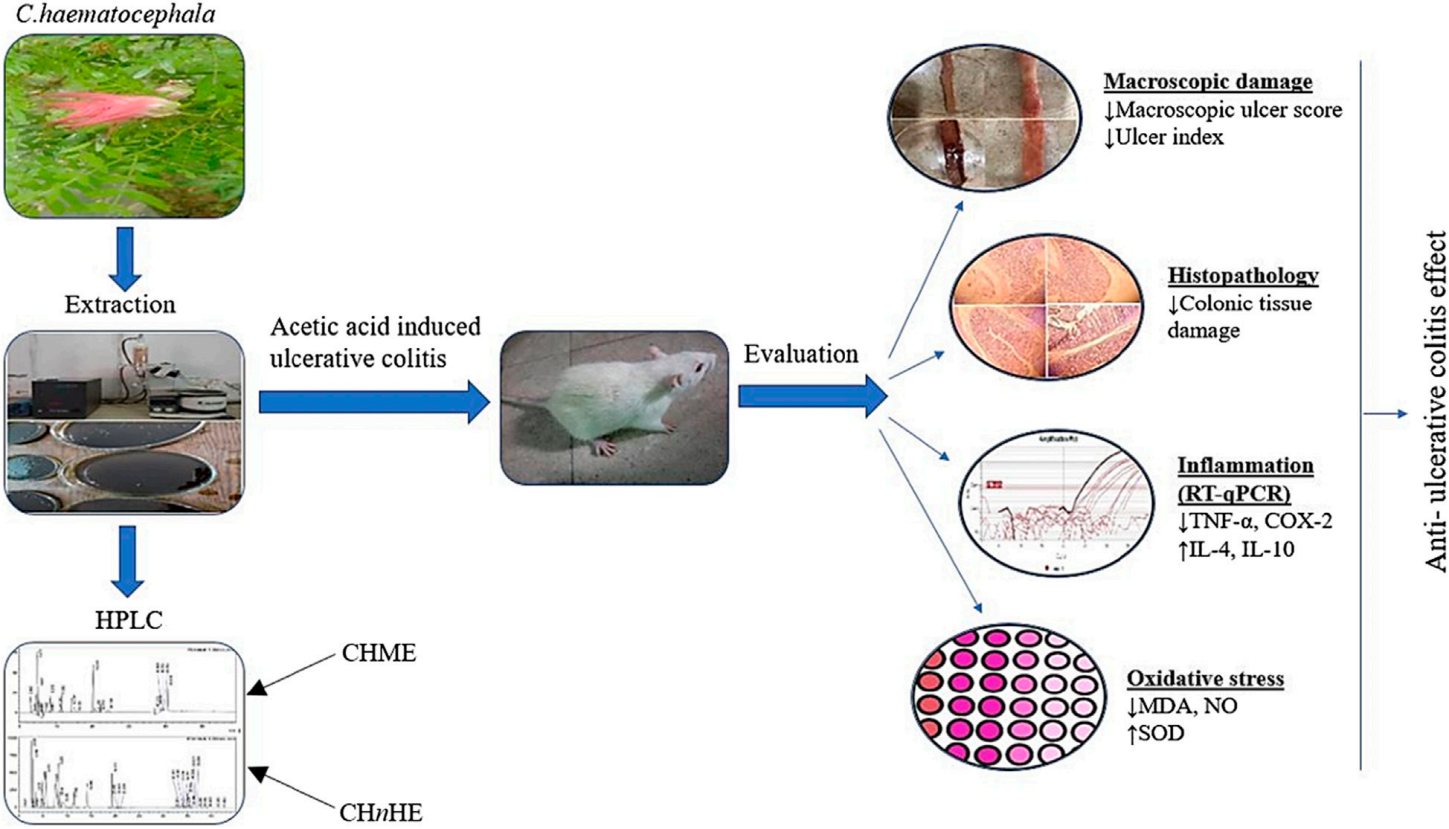
The figure shows graphical overview of study.

The pharmacological effect of *C. haematocephala* in ulcerative colitis has not been elucidated yet despite reports on its gastroprotective activity. In the present study, the anti-ulcerative colitis activity of n-Hexane and methanolic extracts of *C. haematocephala*has been evaluated in a rat model of acetic acid-induced ulcerative colitis. The underlying cellular and molecular mechanisms of anti-ulcerative colitis activity have also been explored in this study. Alongside, the study also covered phytochemical analysis of plant extracts including qualitative and quantitative evaluation to identify the pharmacologically active phytochemicals. The graphical overview of the studyis presented in Figure 1.

## 2 Materials and methods

### 2.1 Plant collection and extraction

*C. haematocephala* was acquired from a plant nursery in Lahore, Pakistan, and was expertly identified by professor of botany, Zaheer-Ud-Din, Government College University, Lahore. The plant was assigned the voucher number GC. Herb. Bot. 3802 and a representative sample was preserved in the university’s herbarium for future reference.

To prepare the methanolic extract (CHME) of *C. haematocephala*, leaves of the plant were washed, shade-dried, and then coarsely powdered by a pulverizer. The powdered leaves were subjected to cold extraction at room temperature using 95% methanol for 6 days with repeated shaking. The extract was filtered first through a cotton cloth and then refined using Whatman filter paper. The excess of solvent was evaporated under reduced pressure and 40°*C* to 45°*C* temperature using a rotary evaporator. The resulting concentrated extract was stored until it was needed for use (Abdul et al., 2010). A similar method was applied for extraction with n-hexane and the extract was concentrated under reduced pressure and temperature in the rotary evaporator (Musthapa et al., 2018).

### 2.2 Qualitative phytochemical analysis

Already established methods were used for qualitative analysis of various phytochemicals including phenols, flavonoids, alkaloids, terpenoids, sterols, tannins, carbohydrates, glycosides, and proteins (Shaikh and Patil, 2020).

### 2.3 Total phenolic content (TPC) and total flavonoid content (TFC) analysis

For the estimation of total phenolic content (TPC), the Folin-Ciocalteu assay was used. Briefly, 1.5 mL of Folin-Ciocalteu reagent was added to 300 µL of plant extract followed by the addition of 1.2 mL of sodium carbonate (7.5%, w/v) in triplicate. Afterward, the mixture was incubated for 30 min in the dark at room temperature. Following the incubation, absorbance was recorded at 765 nm in a UV-visible spectrophotometer. Gallic acid was used for constructing the standard curve. A stock solution of pure gallic acid in distilled water at 100 mg/1,000 mL concentration was prepared for making a set of dilutions at 1, 20, 40, 60, 80, and 100 mg concentration. TPC was described as milligram gallic acid equivalent (mg GAE/g) (Nadiah and Uthumporn, 2015).

Total flavonoid content (TFC) was estimated using an aluminum chloride assay. A standard curve was constructed using quercetin in the concentration range of 0.5–8 μg/mL and TFC was expressed as milligram quercetin equivalent per Gram of dry plant (mg QE/g) (Mahmoudi et al., 2016).

### 2.4 High-performance liquid chromatography (HPLC) analysis

Previously established method of gradient HPLC was used for quantifying the phenolic compounds (Qayyum et al., 2016) and flavonoids (Park et al., 2016) in both plant extracts. Plant extracts were dissolved in 40 mL of 60% methanol followed by acidification with HCl and then heated for 2 h at 90°C. After filtering with 0.2 µm syringe filter, the solutions were injected into reverse phase HPLC that was equipped with C18 columns and a UV-visible detector. Mobile phase is comprised of acetic acid (solvent A) and acetonitrile (solvent B). Initial composition of mobile phase was selected to make the strength appropriate for retaining and resolving the early eluting analytes and afterwards the mobile phase composition was kept on changing and the final composition was selected to ensure the elution of all phytochemicals of interest within reasonable time. Gradient used in HPLC was 15% solvent B for 0–15 min, 45% solvent B for 15–30 min, 100% solvent B for 30–45 mintues. Penolic compounds and flavonoids were identified by their retention times corresponding to UV-visible spectra of peaks and were quantified by a built-in automated system of HPLC.

### 2.5 Experimental animals

Female and male Wistar rats were used in this study. All rats were retained in polypropylene cages and housed in the animal house of the College of Pharmacy, University of the Punjab, Lahore, Pakistan. A twelve-hour light/dark cycle was maintained using artificial light at 50%–60% humidity and 22°*C (±3)* temperature. Rats were given animal food and water*.* The care and handling of the experimental rats followed the guidelines of the National Institute of Health (NIH Publication 85-23, updated in 2002) and were approved by the University’s Ethical Committees at the University of the Punjab in Lahore. Rats were randomly classified into nine different groups, each group consisting of six animals (*n* = 6). The study was initiated from 1st day and continued till 11th day. Group1, labeled as the normal control group (NCG), and GroupII, designated as the colitis control group (CCG), received distilled water daily throughout the study period. GroupIII was the standard control group (SCG) that received 2 mg/kg dose of prednisone daily as reference treatment. To Group-4, Group-5 and Group-6, methanolic extract of *C. haematocephala* (CHME) was administered daily throughout the study period at doses of 250, 500, and 750 mg/kg respectively. Similarly, Group-7, Group-8, and Group-9 received the n-hexane extract of *C. haematocephala* (CHnHE) daily at 250, 500, and 750 mg/kg doses respectively. On the eighth day, ulcerative colitis was induced in all groups except NCG by trans-rectal administration of 1 mL of 4% acetic acid solution after 24 h of fasting. The administration of prednisone, methanolic, and n-hexane extracts was continued in respective groups till the 11th day (Atta et al., 2019).

### 2.6 Assessment of anti-ulcerative colitis activity

The curative potential of *C. ahaematocephala*in ulcerative colitis was evaluated by assessing macroscopic damage, ulcer index, and biochemical, histopathological, pro-inflammatory, and anti-inflammatory parameters. For these assessments, rats were euthanized on 11th day of the study. About 8 cm of colon 2 cm proximal to the anus was removed and washed with normal saline (Atta et al., 2019). It was mounted on a glass slide for macroscopic damage evaluation and was photographed. Immediately afterward, the colon specimen was divided into two portions. One portion was fixed in 10% formaldehyde for histopathological examination and the other portion was stored at −20°C for biochemical analysis. Blood samples of rats were collected by cardiac puncture to evaluate the mRNA expression of pro-inflammatory and anti-inflammatory mediators using RT-qPCR.

### 2.7 Assessment of colonic macroscopic damage

Macroscopic damage was assessed on the basis of macroscopic ulcer score and ulcer index which were measured by affected area of colon to demonstrate the severity and extent of colon tissue damage induced by acetic acid.

### 2.8 Assessement of macroscopic ulcer score

For scoring of macroscopic damage, isolated colon piece of 8 cm long was mounted on a slide. Macroscopic ulcer score depicted the severity of colon damage and was evaluated by a score scale established by Miller that was based on clinical features as shown in Table 1. All these scores were scaled blindly after cautious observation of colon (Adakudugu et al., 2020).

**TABLE 1:** Scale bar for quantification of macroscopic ulcer score in acetic acid induced ulcerative colitis.

Macroscopic ulcer score	Severity of colon lesion
0	No macroscopic change
1	Only mucosal erythema
2	Small erosions or minor bleeding with mild edema
3	Small bleeding erosions with moderate edema
4	Tissue necrosis, severe ulceration and edema

### 2.9 Assessment of ulcer index (UI)

The extent of colon damage induced by acetic acid was assessed by ulcer index (UI) which was calculated by dividing the ulcer area of the colon (sq. mm) by the total area of the colon (sq. mm); Ulcer index (UI) = $\frac{\text{Ulcer}\;\text{area}\;\text{of}\;\text{colon}\;\left(\text{sq}.\;\text{mm}\right)}{\text{Total}\;\text{area}\;\text{of}\;\text{colon}\;\left(\text{sq}.\;\text{mm}\right)}$

### 2.10 Measurement of colonic malondialdehyde (MDA) content

The level of colonic MDA (malondialdehyde) in colon tissues for assessing oxidative stress was determined by an already-established method. Accordingly, 100 µL of tissue homogenate was mixed with 2.5 mL reaction buffer (15% trichloroacetic acid, 0.25 M HCl, and 0.37% thiobarbituric acid in 1:1:1 ratio) and the mixture was heated 95°C for 60 min. After that, the mixture was cooled and centrifuged for 10 min at 3,500 rpm. Lastly, the absorbance of the supernatant was measured at 535 nm. MDA content was measured as nM of MDA/g of tissue using a standard curve (Froushani and Mashouri, 2018; Asgharzadeh et al., 2021).

### 2.11 Measurement of colonic nitrite/nitrate level

For the measurement of nitric oxide in colon specimen to analyze oxidative stress, a modified protocol of Griess assay was used. Briefly, 50 µL of Griess reagent comprising phosphoric acid, sulfanilamide and N-1-naphthylethylenediaminewas mixed with 50 µL of sample supernatant in a 96-well plate. The reaction mixture was shaken for 10 min in a dark room and absorbance was found at 540 nm against blank. Sodium nitrite was used as a reference standard for making a calibration curve which was then used for nitric oxide quantification as nmol/g of tissue (Siddiqua et al., 2022).

### 2.12 Estimation of superoxide dismutase (SOD)

The supernatant of colon tissue was used for the measurement of SOD using SOD assay kit according to the protocol given by the manufacturer (Tang et al., 2021). Measurement of SOD activity was done to assess the functionality of the antioxidant defense system and the balance between oxidant and antioxidant mechanisms.

### 2.13 Histopathological examination

On very next day after sacrificing the rats, preserved colon samples were processed for microscopic examination by washing them with tap water and dehydrating them with serial dilutions of ethanol. The samples were then cleared in xylene and embedded in paraffin. The paraffin tissue blocks were cut into thin sections (5 microns) using a slide microtome and mounted on glass slides. The tissue sections were deparaffinized and stained with hematoxylin and eosin (H&E) before being examined under a light microscope using ×40 magnification (Suvarna et al., 2018). Histopathological examination was evaluated by an independent pathologist who was blinded for treatments and was assessed based on inflammatory changes, cellular infiltration, and colon thickening. The evaluation was measured on an ascending scale of 0–4 as shown in Table 2 (Badr et al., 2020).

**TABLE 2:** Histopathological scale quantification in acetic acid induced ulcerative colitis.

Histopathological score	Microscopic cellular and inflammatory changes
0	No inflammatory evidence
1	Mild inflammation with 1–2 foci of mononuclear cell infiltration
2	Moderate inflammation accompanying multiple foci
3	Severe inflammation with marked thickening of colon wall
4	Severe inflammation with loss of goblet cells and transmural leukocyte infiltration

### 2.14 Quantification of pro and anti-inflammatory markers by RT-qPCR

RT-qPCR was used for evaluating the gene expression of pro and anti-inflammatory markers including TNF-α, COX-2, IL-4 and IL-10. For this purpose, RNA was extracted using a Pure-link RNA mini kit (Invitrogen; catalogue# 12183018A). Isolated samples of RNA were qualified, quantified and then equalized using NanoDrop quantification. Then by using 10 ng/μL of RNA as minimum input, cDNA was synthesized through reverse transcription using Revert Aid First Strand cDNA synthesis kit (catalogue#: 4472903) acquired from Thermo Fisher Scientific Services. Although, cDNA can be stored for years at −20°C in Tris-EDTA buffer pH 8.0, however in current study, the cDNA was subjected to RT-qPCR after 1 day of its synthesis. By using relevant primers, the cDNA was taken as a template for carrying out the real-time gene expression of pro and anti-inflammatory cytokines using SYBER Select Master Mix (catalogue#: 4472903). The sequence of temperature conditions with corresponding time intervals during the process of RT-qPCR was set as 50°C for 2 min, 95°C for 10 min, 95°C for 15 s, and 60°C for 1 min. GAPDH was used as an endogenous control for normalizing the relative quantification of targeted gene expression. For quantifying the level of gene expression of pro and anti-inflammatory genes, 2^−ΔΔCT^ method was applied, where CT is cycle threshold (Wang et al., 2016). TNF-α and COX-2 were the pro-inflammatory genes quantified by RT-qPCR whereas the anti-inflammatory genes were IL-4 and IL-10. Primers used for genes have been shown in Table 3.

**TABLE 3:** Primers used for mRNA expression analysis of pro and anti-inflammatory markers by RT-qPCR.

Markers	Forward/Reverse	Sequence	Gene accession
TNF-α	Forward	5′-ATG​GGC​TCC​CTC​TCA​TCA​GT-3′	NM 012675.3
	Reverse	5′-GCTTGGTGGTTTGCT ACGA.C-3′	
COX-2	Forward	5′-A TGC​TAC​CAT​CTG​GCT​TCG​G-3′	NM 017232.3
	Reverse	5′-TGGAACA GTCGCTCGTCATC-3′	
IL-4	Forward	5′-GTA​CCG​GGA​ACG​GTA​TCC​AC-3′	NM 201270.1
	Reverse	5′-TGGTGTTCCTTGTTG CCGTA-3′	
IL-10	Forward	5′-TTG​AAC​CAC​CCG​GCA​TCT​AC-3′	NM 012854.2
	Reverse	5′-CCAAGGA GTTGCTCCCGTTA-3′	

### 2.15 Statistical analysis

Data was expressed as mean ± standard deviation (SD) and was analyzed statistically by one-way ANOVA followed by *post hoc* Tukey’s test wherein applicable using Graph pad prism 8.0 software. The significance of the difference in results among groups was assessed based on “*p”* value as; *p* < 0.05 represents significant difference, *p* < 0.01 represents very significant difference, and *p* < 0.001 represents highly significant difference. The alphabets “a”, “b,” and “c” were used to exhibit the significant difference of treatment groups from CCG, NCG, and SCG respectively.

## 3 Results

### 3.1 Qualitative phytochemical analysis

The phytochemicals identified in this study, including polyphenols, flavonoids, tannins, alkaloids, and sterols were found in CHME and CH*n*HE through qualitative analysis. Notably, primary metabolites such as carbohydrates, proteins, and glycosides were absent. This analysis suggests that the presence of these secondary metabolites have the potential to exert biological effects.

### 3.2 Total phenolic content (TPC) and total flavonoid content (TFC) analysis

Furthermore, the study quantified total phenolic content (TPC) and total flavonoid content (TFC) and found higher TPC in CH*n*HE (79.16 ± 1.30 mg GAE/g) than CHME (63.31 ± 0.81 mg GAE/g). Similarly, higher TFC was found in CHnHE (61.01 ± 0.94 mg GCE/g) than CHME (53.33 ± 0.11 mg GAE/g). These findings suggest that the anti-ulcerative colitis potential of CH*n*HE and CHME may be attributed to the significant TPC and TFC in these extracts. These secondary metabolites have been reported to downregulate the mRNA expression of pro-inflammatory mediators and upregulate the mRNA expression of anti-inflammatory mediators.

### 3.3 High performance liquid chromatography (HPLC) analysis

**FIGURE 2:**
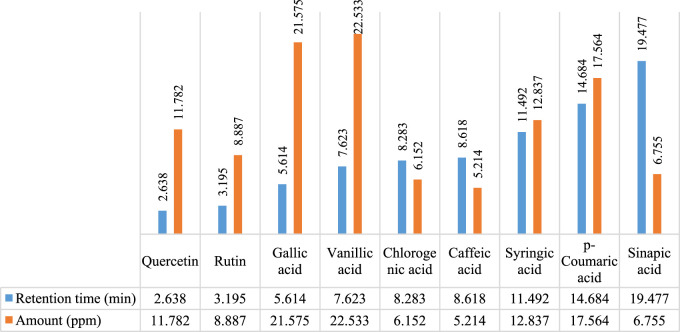
HPLC quantification of flavonoids and polyphenols in CH*n*HE. Figure shows the relative quantities of flavonoids (quercetin and rutin) and polyphenols (gallic acid, vanillic acid, chlorogenic acid, caffeic acid, syringic acid, *p*-coumaric acid, sinapic acid) in CHnHE. Bars in blue color represent the retention times of all phytochemicals whereas red colored bars represent the corresponding concentrations of these identified compounds.

**FIGURE 3:**
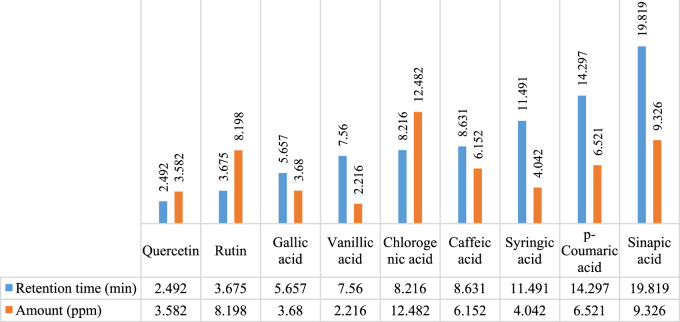
HPLC quantification of flavonoids and polyphenols in CHME. Figure shows the relative quantities of flavonoids (quercetin and rutin) and polyphenols (gallic acid, vanillic acid, chlorogenic acid, caffeic acid, syringic acid, *p*-coumaric acid, sinapic acid) in CHME. Bars in blue color represent the retention times of all phytochemicals whereas red colored bars represent the corresponding concentrations of these identified compounds.

**FIGURE 4:**
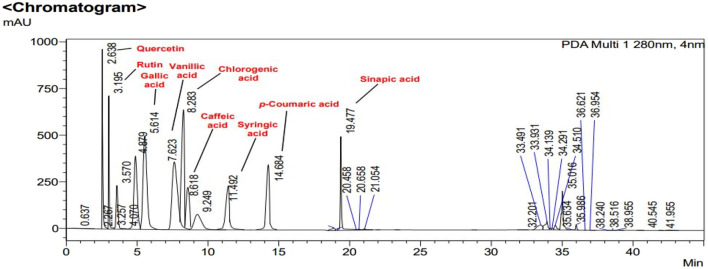
HPLC chromatogram of CH*n*HE. Peaks in chromatogram represent the concentrations (ppm) of flavonoid and polyphenolic compounds according to their retention times (minutes).

**FIGURE 5:**
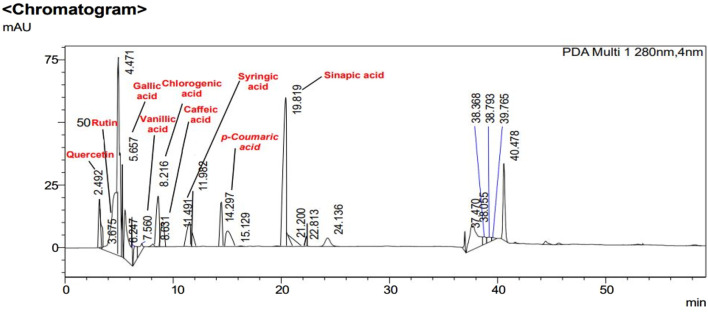
HPLC chromatogram of CHME. Peaks in chromatogram represent the concentrations (ppm) of flavonoid and polyphenolic compounds according to their retention times (minutes).

In Figures 2, 3, concentrations of various flavonoids and polyphenols in CH*n*HE and CHME quantified by HPLC have been shown which were measured according to retention times corresponding to their conentrations. Corresponding HPLC peaks of CH*n*HE and CHME are shown in Figures 4, 5 respectively.

### 3.4 Effect on colonic macroscopic udamage

**FIGURE 6:**
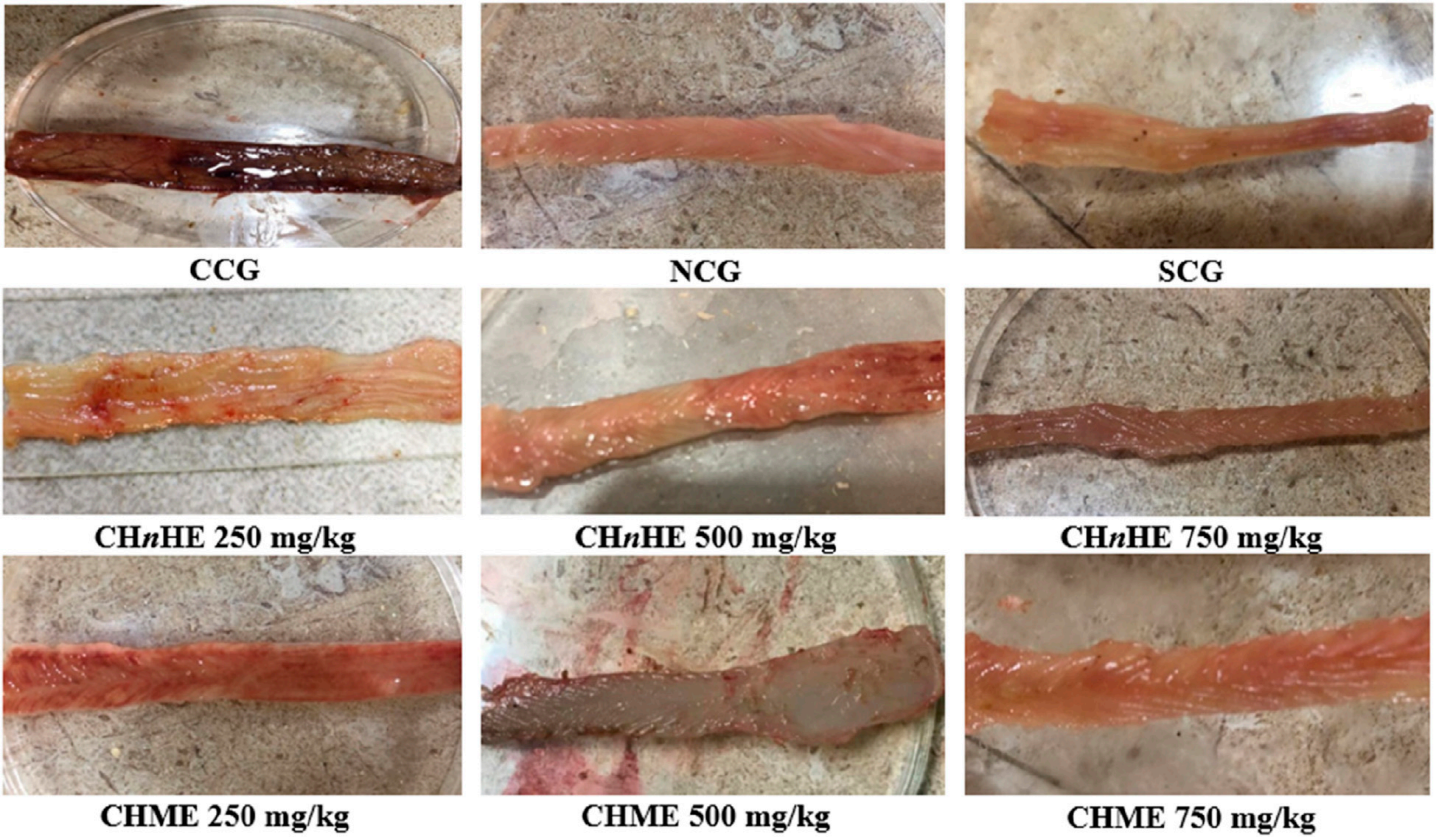
Effect of plant extracts on colon tissues. Figure shows the macroscopic observation of damage in the colon of acetic acid-induced ulcerative colitis after treatment with CH*n*HE and CHME extracts at 250, 500, and 750 mg/kg doses. Damaged area was used to calculate macroscopic ulcer score and ulcer index. Figure shows a progressive decrease in tissue damage by plant extracts and SCG with increase in dose in contrast to CCG that shows remarkable tissue damage. Here, CCG, NCG, and SCG represent “colitis control group,” “normal control group,” and “standard control group” respectively.

Figure 6 is depicting the macroscopic damage observable macroscopically. This macroscopic damage was evaluated in terms of macroscopic ulcer score and ulcer index which were quantified from this damage. Macroscopic ulcer score represented the severity of colon tissue damage and ulcer index represented the extent of colon tissue damage. As shown in Figure 6, NCG, SCG, 250, 500, and 750 mg/kg doses of CH*n*HE and CHME depicted significantly lower macroscopic damage as compared to CCG.

### 3.5 Effect on macroscopic ulcer score

**FIGURE 7:**
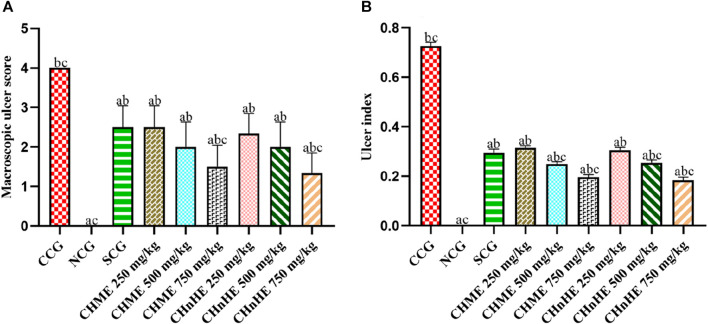
Effect of *C. haematocephala* extracts (CH*n*HE and CHME) on macroscopic ulcer score **(A)** and ulcer index **(B)** in acetic acid induced ulcerative colitis. Figure shows significant dose dependent decline in macroscopic ulcer score and ulcer index after treatment for consective 11 days with CHnHE and CHME (250, 500, and 750 mg/kg doses) standardized with prednisone (2 mg/kg) in comparison (*p* < 0.001) to CCG. One-way ANOVA followed by *post hoc* Tukey’s test was applied to analyze the significance of differences among all treatment and control groups. Here, “a,” “b,” and “c” indicate significant differences from CCG, NCG, and SCG respectively.

As shown in Figure 7A, significant decrease in macroscopic ulcer score was exhibited by CH*n*HE at 250 mg/kg (2.3 ± 0.5), 500 mg/kg (2.0 ± 0.6), and 750 mg/kg (1.3 ± 0.5) doses, CHME at 250 mg/kg (2.5 ± 0.5), 500 mg/kg (2.0 ± 0.6), and 750 mg/kg (1.5 ± 0.5) doses, SCG (2.5 ± 0.5), and NCG (0 ± 0) in contrast (*p* < 0.001) to CCG (4.0 ± 0). Decrease in macroscopic ulcer score by both extracts was dose dependent and highest decrease among all treatments was shown by 750 mg/kg dose of CH*n*HE. Effects shown by 250 mg/kg and 500 mg/kg doses of both extracts were comparable (*p* > 0.05) to SCG whereas 750 mg/kg doses of CH*n*HE (*p* < 0.01) and CHME (*p* < 0.05) decreased the macroscopic ulcer score significantly higher as compared to SCG.

### 3.6 Effect on ulcer index

Figure 7B is depicting significant decrease in ulcer index by CH*n*HE at 250 mg/kg (0.30 ± 0.01), 500 mg/kg (0.25 ± 0.01), and 750 mg/kg (0.18 ± 0.01) doses, CHME at 250 mg/kg (0.31 ± 0.00), 500 mg/kg (0.25 ± 0.01), and 750 mg/kg (0.19 ± 0.01) doses, SCG (0.29 ± 0.01), and NCG (0.00 ± 0.00) in contrast (*p* < 0.001) to CCG (0.72 ± 0.01). Similar to the pattern observed in macrsoscopic damage score, decrease in ulcer index by both extracts was also dose dependent and highest decrease among all treatments was shown by 750 mg/kg dose of CH*n*HE. Effects shown on ulcer index by 250 mg/kg doses of both extracts were comparable (*p* > 0.05) to SCG whereas 500 mg/kg and 750 mg/kg doses of both extracts decreased the ulcer index significantly higher (*p* < 0.001) as compared to SCG.

### 3.7 Effect on oxidative stress

**FIGURE 8:**
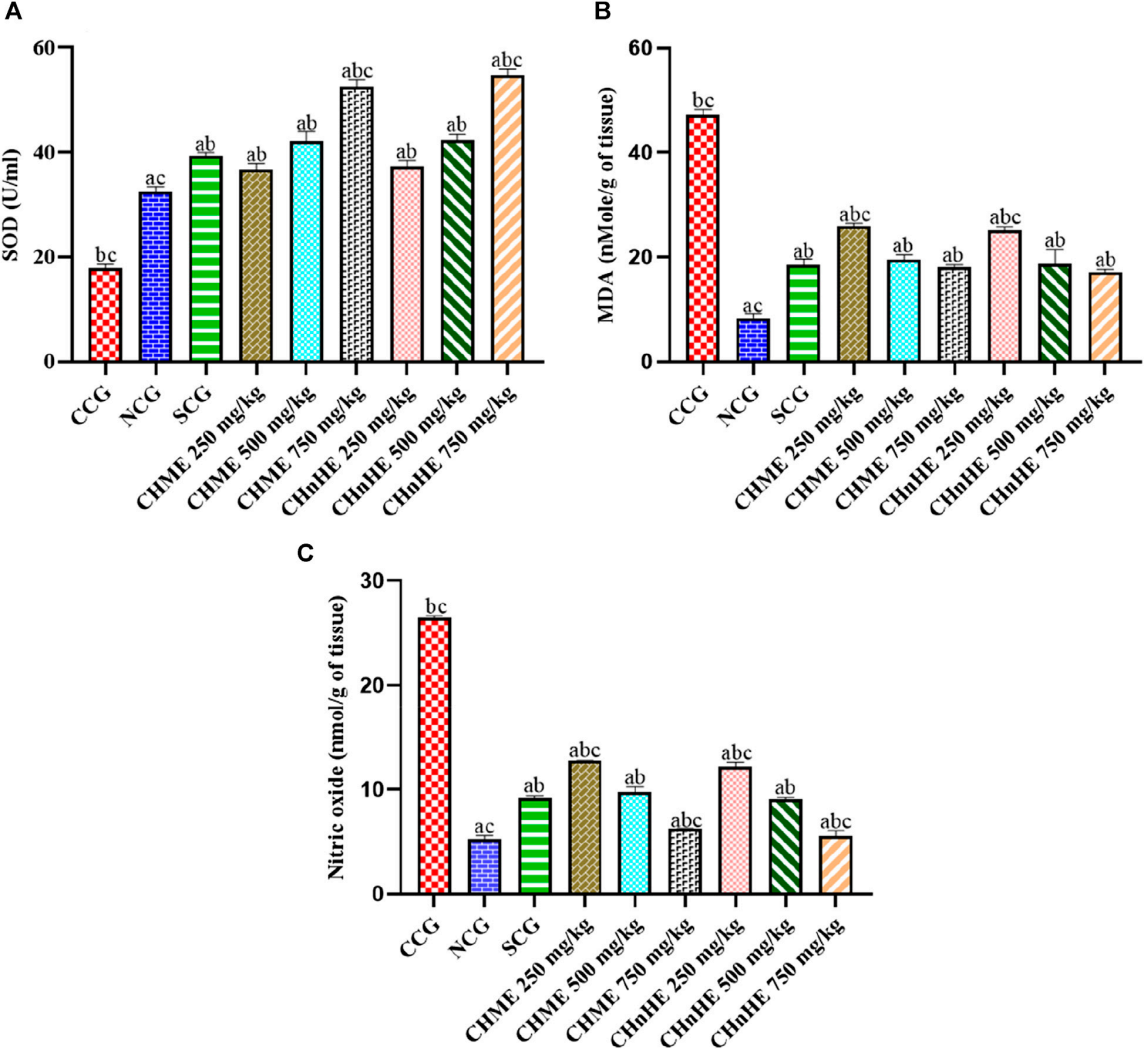
Effect of *C. haematocephala* extracts (CH*n*HE and CHME) on oxidative stress markers; SOD **(A)**, MDA **(B)**, and NO **(C)** in acetic acid induced ulcerative colitis. Figure shows significant dose dependent increase in SOD activity and decline in MDA and NO synthesis after treatment for consective 11 days with CHnHE and CHME (250, 500, and 750 mg/kg doses) standardized with prednisone (2 mg/kg) in comparison (*p* < 0.001) to CCG. One-way ANOVA followed by posthoc Tukey’s test was applied to analyze the significance of differences among all treatment and control groups. Here, “a,” “b,” and “c” indicate significant differences from CCG, NCG, and SCG respectively.

Both extracts and SCG decreased the oxidative stress by increasing the activity of antioxidant enzyme superoxide dismutase (SOD) and decreasing the production of oxidative species malondialdehyde (MDA) and nitric oxide (NO) in contrast to CCG. As shown in Figure 8A, significant increase in SOD activity was exhibited by CH*n*HE at 250 mg/kg (37.27 ± 1.16 U/mL), 500 mg/kg (42.39 ± 1.13 U/mL), and 750 mg/kg (54.72 ± 1.19 U/mL) doses, CHME at 250 mg/kg (36.71 ± 1.18 U/mL), 500 mg/kg (42.22 ± 1.81 U/mL), and 750 mg/kg (52.63 ± 1.22 U/mL) doses, SCG (39.43 ± 0.52 U/mL), and NCG (32.58 ± 0.84 U/mL) in contrast (*p* < 0.001) to CCG (17.97 ± 0.71 U/mL). Increase in SOD activity by both extracts was dose dependent and highest increase among all treatments was shown by 750 mg/kg dose of CH*n*HE. Increase in SOD activity shown by 250 mg/kg and 500 mg/kg doses of both extracts were comparable (*p* > 0.05) to SCG whereas 750 mg/kg doses of CH*n*HE and CHME increased the SOD activity significantly higher (*p* < 0.001) as compared to SCG.

A significant (*p* < 0.001) decrease in colon tissue level of MDA, an oxidative marker, was observed in CH*n*HE at 250 mg/kg (25.23 ± 0.57 nMol/g of tissue), 500 mg/kg (18.76 ± 2.77 nMol/g of tissue), and 750 mg/kg (17.23 ± 0.50 nMol/g of tissue) doses, CHME at 250 mg/kg (25.90 ± 0.64 nMol/g of tissue), 500 mg/kg (19.57 ± 0.93 nMol/g of tissue), and 750 mg/kg (18.14 ± 0.43 nMol/g of tissue) doses, SCG (18.47 ± 1.18 nMol/g of tissue), and NCG (8.42 ± 0.79 nMol/g of tissue) in contrast (*p* < 0.001) to CCG (47.19 ± 1.07 nMol/g of tissue) as shown in Figure 8B. Decrease in MDA level by both extracts was dose dependent and highest decrease among all treatments was shown by 750 mg/kg dose of CH*n*HE. Decrease in MDA level shown by 500 mg/kg and 750 mg/kg doses of both extracts were comparable (*p* > 0.05) to SCG.

Figure 8C is depicting the significant decrease in level of another oxidative species nitric NO in colon tissues by treatment with CH*n*HE at 250 mg/kg (12.15 ± 0.49 nMol/g of tissue), 500 mg/kg (9.06 ± 0.16 nMol/g of tissue), and 750 mg/kg (5.51 ± 0.54 nMol/g of tissue) doses, CHME at 250 mg/kg (12.73 ± 0.06 nMol/g of tissue), 500 mg/kg (9.75 ± 0.49 nMol/g of tissue), and 750 mg/kg (6.20 ± 0.06 nMol/g of tissue) doses, SCG (9.17 ± 0.22 nMol/g of tissue), and NCG (5.30 ± 0.29 nMol/g of tissue) in contrast (*p* < 0.001) to CCG (26.53 ± 0.16 nMol/g of tissue). Decrease in NO level by both extracts was dose dependent and highest decrease among all treatments was shown by 750 mg/kg dose of CH*n*HE. Decrease in NO level shown by 500 mg/kg doses of both extracts were comparable (*p* > 0.05) to SCG whereas 750 mg/kg doses decreased the NO level significantly higher (*p* < 0.001) as compared to SCG.

### 3.8 Histopathological evaluation

**FIGURE 9:**
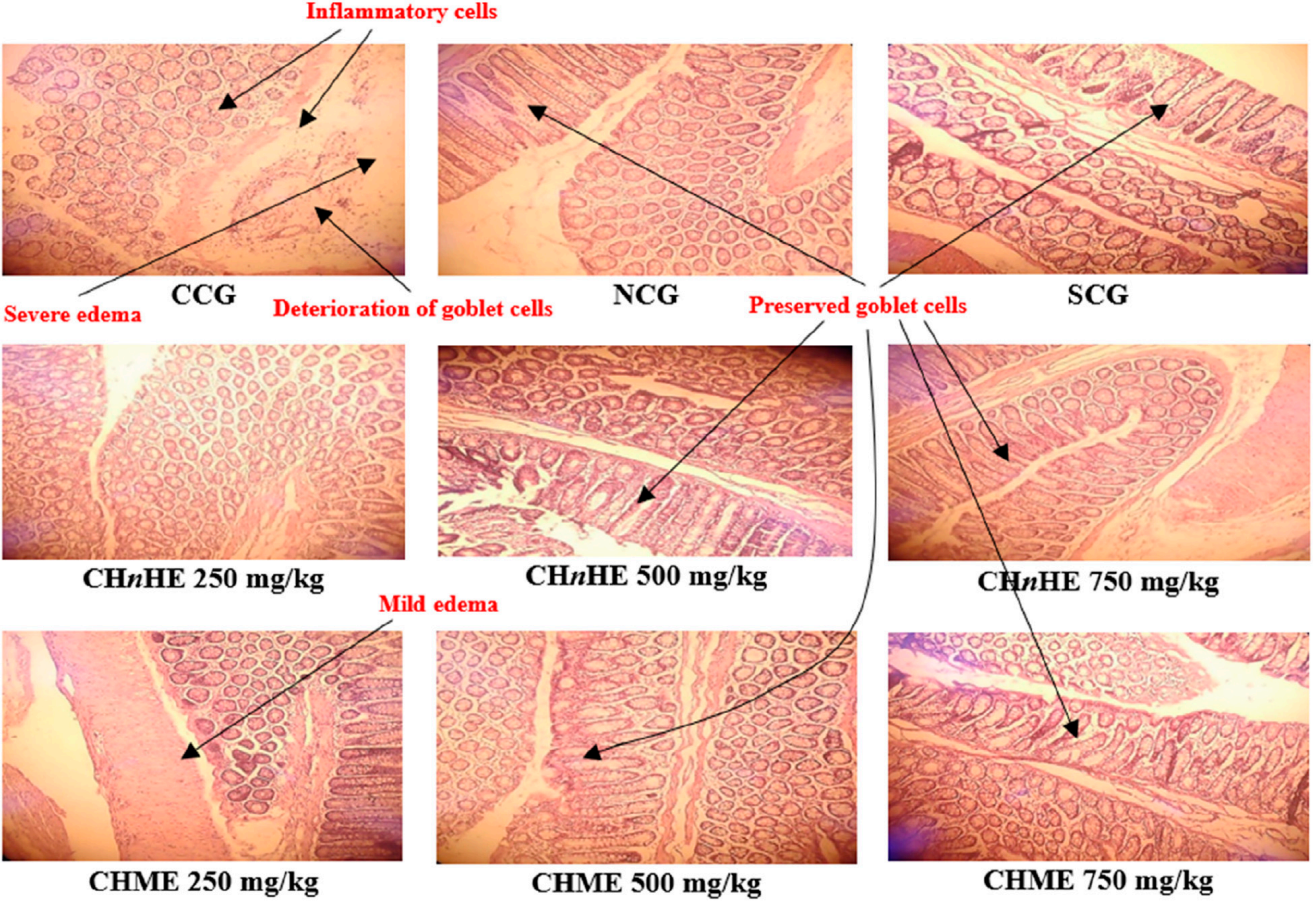
Effect of *C. haematocephala* extracts (CH*n*HE and CHME) on histopathological architecture of colon in acetic acid induced ulcerative colitis. Figure shows histopathological slides representing the cellular and molecular aspects of colon inflammatory damage. All treatment groups and NCG exhibited the intact goblet cells and prevented the infiltration of inflammatory cells and edema in colon tissues. Here, CCG, NCG, and SCG represent “colitis control group,” “normal control group,” and “standard control group” respectively.

**FIGURE 10:**
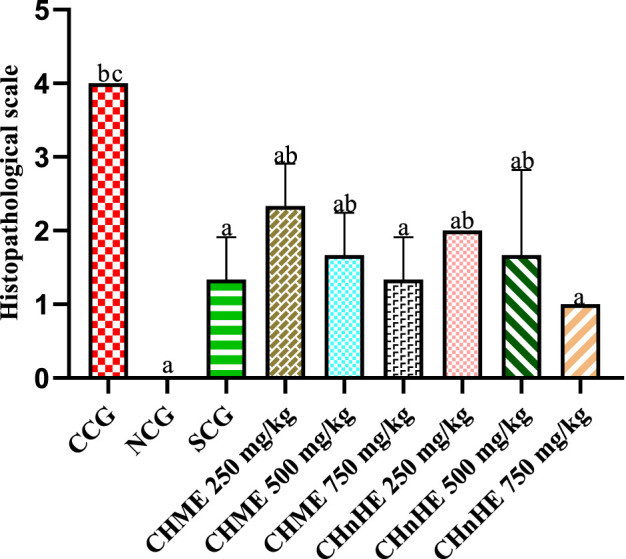
Effect of *C. haematocephala* extracts (CHnHE and CHME) on histopathological scale in acetic acid induced ulcerative colitis. Histopathological scale was quantified by inflammatory changes using histopathological slide examination given in Figure 9. This figure shows dose dependent decrease in histopathological scale after treatment with CHnHE and CHME (250, 500, and 750 mg/kg doses) standardized with prednisone (2 mg/kg) in comparison (*p* < 0.001) to CCG. One-way ANOVA followed by *post hoc* Tukey’s test was appliedto analyze the significance of differences among groups. Here, “a”, “b”, and “c” indicate significant differences from CCG, NCG and SCG respectively.

Histopathological evaluation revealed significant amelioration in inflammation, edema and integrity of goblet cells at all treatment doses of both extracts in addition to SCG and NCG in contrast to CCG as shown in Figure 9. All these pathological features were quantified in terms of already established histopathological scale representing the histopathological damage of colon and a lower scale value represented higher histopathological protection. It was shown in Figure 10 that 750 mg/kg (1.00 ± 0) dose of CH*n*HE exhibited the lowest histopathological scale followed by 750 mg/kg (1.33 ± 0.57) in addition to SCG (1.33 ± 0.57) and NCG (0 ± 0) and all exhibited significantly lower scale values in contrast (*p* < 0.001) to CCG (4.00 ± 0). Histopathological scale exhibited by CH*n*HE at 500 mg/kg (1.66 ± 0.57) and 250 mg/kg (2.00 ± 0) doses and CHME at 500 mg/kg (1.66 ± 0.57) dose was also significantly lower (*p* < 0.01) in contrast to CCG. 250 mg/kg (2.3 ± 0.57) dose of CHME exhibited the least decline in histopathological scale among all treatment groups but it was significantly lower (*p* < 0.01) as compared to CCG. Decrease in histopathological scale by all doses of both extracts was comparable (*p* > 0.05) to SCG.

### 3.9 Effect on mRNA expression of pro and anti-inflammatory mediators

**FIGURE 11:**
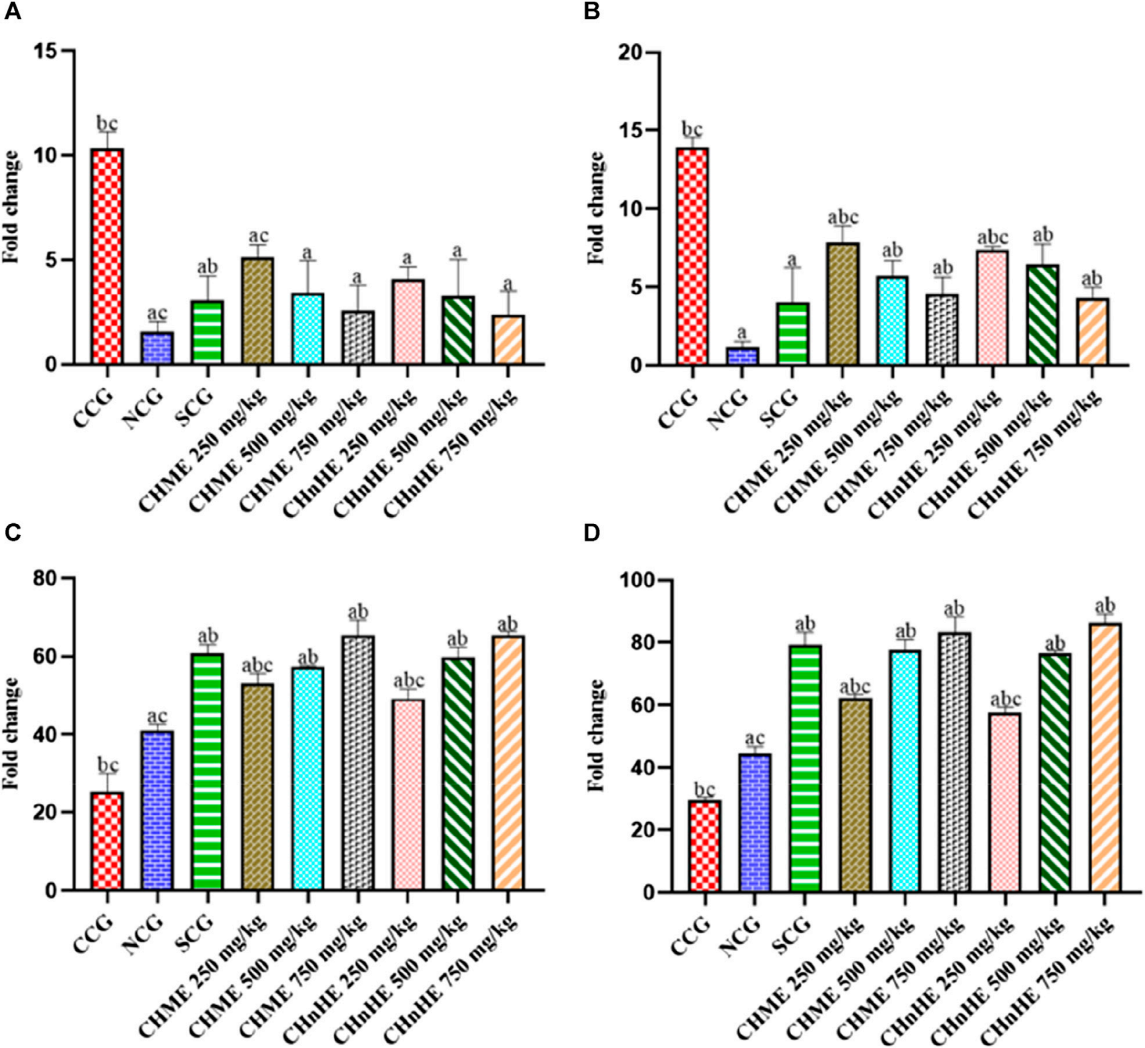
Effect of *C. haematocephala* extracts (CH*n*HE and CHME) on mRNA expression of pro and anti-inflammatory mediators; TNF-α **(A)**, COX-2 **(B)**, IL-4 **(C)**, and IL-10 **(D)** in acetic acid induced ulcerative colitis. mRNA expression of all these mediators was normalized using GAPDH as housekeeping gene. Figure shows significant downregulation of mRNA expression of TNF-α cytokine and COX-2 enzyme whereas upregulation of mRNA expression of IL-4 and IL-10 after treatment for consective 11 days with CH*n*HE and CHME (250, 500, and 750 mg/kg doses) standardized with prednisone (2 mg/kg) in comparison (*p* < 0.001) to CCG. One-way ANOVA followed by *post hoc* Tukey’s test was appliedto analyze the significance of differences among all treatment and control groups. Here, “a,” “b,” and “c” indicate significant differences from CCG, NCG, and SCG respectively.

mRNA expression analyzed at the end of study perioid after sacrificing the rats revealed significant downregulation of pro-inflammatory cytokines and upregulation of anti-inflammatory cytokines by all doses of both extracts and SCG in addition to NCG in contrast to CCG as shown in Figure 11. This downregulation of pro-inflammatory mediators and upregulation of anti-inflammatory cytokines represented the disease modifying otential of both plant extracts. As shown in Figure 11A, significant downregulation of TNF-α mRNA was exghibited by CH*n*HE at 250 mg/kg (4.08 ± 0.58 fold change), 500 mg/kg (3.29 ± 1.74 fold change), and 750 mg/kg (2.37 ± 1.13 fold change) doses, CHME at 250 mg/kg (5.15 ± 0.56 fold change), 500 mg/kg (3.42 ± 1.56 fold change), and 750 mg/kg (2.57 ± 1.21 fold change) doses, SCG (3.09 ± 1.14 fold change), and NCG (1.56 ± 0.49 fold change) in contrast (*p* < 0.001) to CCG (10.31 ± 0.82 fold change). Both extracts downregulated the TNF-α mRNA in dose dependent manner and 750 mg/kg dose of CH*n*HE exhibited the downregulation to highest extent. Downregulation of TNF-α mRNA by all doses of both extracts was comparable (*p* > 0.05) to SCG.

As shown in Figure 11B, significant downregulation of COX-2 mRNA expression was exhibited by CH*n*HE at 250 mg/kg (7.39 ± 0.19 fold change), 500 mg/kg (6.47 ± 1.26 fold change), and 750 mg/kg (4.33 ± 0.65 fold change) doses, CHME at 250 mg/kg (7.82 ± 1.08 fold change), 500 mg/kg (5.69 ± 1.01 fold change), and 750 mg/kg (4.56 ± 1.07 fold change) doses, SCG (4.06 ± 2.18 fold change), and NCG (1.19 ± 0.33 fold change) in contrast (*p* < 0.001) to CCG (13.91 ± 0.64 fold change). Both extracts downregulated the COX-2 mRNA in dose dependent manner and 750 mg/kg dose of CH*n*HE exhibited the downregulation to highest extent. Downregulation of COX-2 mRNA by 500 mg/kg and 750 mg/kg doses of both extracts was comparable (*p* > 0.05) to SCG.

Significant upregulation of IL-4 mRNA expression was exhibited by CH*n*HE at 250 mg/kg (53.10 ± 2.50 fold change), 500 mg/kg (59.73 ± 2.64 fold change), and 750 mg/kg (65.53 ± 0.89 fold change) doses, CHME at 250 mg/kg (49.26 ± 2.41 fold change), 500 mg/kg (57.41 ± 0.31 fold change), and 750 mg/kg (65.50 ± 3.72 fold change) doses, SCG (60.79 ± 2.22 fold change), and NCG (41.00 ± 1.62 fold change) in contrast (*p* < 0.001) to CCG (25.36 ± 4.72 fold change) as shown in Figure 11C. Both extracts upregulated the IL-4 mRNA in dose dependent manner and 750 mg/kg dose of CH*n*HE exhibited the upregulation to highest extent. Upregulation of IL-4 mRNA by 500 and 750 mg/kg doses of both extracts was comparable (*p* > 0.05) to SCG.

Significant upregulation of IL-10 mRNA expression was exhibited by CH*n*HE at 250 mg/kg (57.60 ± 1.89 fold change), 500 mg/kg (76.50 ± 0.97 fold change), and (86.28 ± 2.91 fold change) doses, CHME at 250 mg/kg (62.29 ± 1.13 fold change), 500 mg/kg (77.56 ± 3.28 fold change), and 750 mg/kg (83.29 ± 4.99 fold change) doses, SCG (79.33 ± 3.99 fold change), and NCG (44.60 ± 2.15 fold change) in contrast (*p* < 0.001) to CCG (29.64 ± 0.71 fold change) as shown in Figure 11D. Both extracts upregulated the IL-10 mRNA in dose dependent manner and 750 mg/kg dose of CH*n*HE exhibited the upregulation to highest extent. Upregulation of IL-10 mRNA by 500 mg/kg and 750 mg/kg doses of both extracts was comparable (*p* > 0.05) to SCG.

## 4 Discussion

Lack of a definite cure, drug resistance, significant adverse effects, response failure, and high economic cost of available drugs encourage the research for curative therapies devoid of these drawbacks. Plant-based medicines are being recognized as a novel panacea by the general public because of fewer side effects and high efficacy (Singh et al., 2020).

This study was designed to explore the anti-ulcerative colitis potential of methanolic (CHME) and *n*-Hexane (CH*n*HE) extracts of *Calliandra haematocephala* along with associated mechanisms. For this purpose, a rat model of ulcerative colitis was used. Induction of ulcerative colitis with acetic acid is a widely used standard experimental method that resembles human ulcerative colitis in several pathophysiological features including inflammation, damage to mucosal and sub-mucosal layers, cellular infiltration, and colon tissue necrosis (Hassanshahi et al., 2020).

Tannins, alkaloids, and sterols detected in CHME and CH*n*HE by qualitative analysis have been reported to have anti-inflammatory activity (Obaseki et al., 2016). Phenolic acids and flavonoids detected and quantified in CHME and CH*n*HE by HPLC can exert anti-inflammatory action that can be attributed to their anti-ulcerative activity as also reported by a previous study on a different plant (Gupta et al., 2018). A single phytoconstituent can probably target multiple pathophysiological pathways and even a single pathological or biochemical pathway can be affected by multiple phytochemicals (Shin et al., 2020). The current study was focused on principal pathophysiological pathways of ulcerative colitis in correlation to the effects of the identified phytochemicals on these pathways.

Alkaloids offer attractive source of drug discover and their role in resolving inflammation and oxidative stress is well established. Alkaloids have the potential to inhibit different pathogenic pathways of inflammation including NF-κB (Bai et al., 2021). Present study reprted the anti-ulcerative colitis effect of CHME and CHnHE that can be attributed to the presence of alkaloids as detected by phytochemical analysis. Macroscopic damage induced by acetic acid in UC is due to overtly produced TNFα that triggers the degradation of the intestinal matrix resulting in colon ulceration via epithelial damage and bleeding due to vascular disruption (Adegbola et al., 2018). Edema observed in macroscopic damage is associated with the overproduction of COX-2 which produces edema via the production of PGE-2 (El-Tanbouly and Abdelrahman, 2022). The apoptotic and necrotic features of UC observable macroscopically occur due to oxidative stress induced by NO along with reactive oxygen species (Shahid et al., 2022). Evaluation of macroscopic damage is used as a valuable tool in the assessment of anti-ulcerative colitis activity of tested treatments (Allodi et al., 2023). Amelioration of colon ulcers and bleeding by CHME and CH*n*HE in a dose-dependent manner as observed in the present study has exhibited the anti-ulcerative colitis effect of *C. haematocephala.* This protective effect may be attributed to *p*-Coumaric acid and chlorogenic acid detected in both extracts as these phenolic acids have been documented to decrease the expression of TNF-α in a previous study (Mohammed et al., 2023). Anti-edematous effect exhibited by CHME and CH*n*HE in this study can be most probably owned by the presence of vanillic acid in the study plant as supported by a previous work in which this phenolic compound revealed down production of COX-2 production (Kaur et al., 2022). Prevention of necrosis exhibited by CHME and CH*n*HE is most probably due to the presence of rutin which has the potential to inhibit NO and has anti-apoptotic activity as documented in some studies (El Menyiy et al., 2022). Associated with the macroscopic assessment of UC, evaluation of anti-ulcerative colitis activity of tested treatments can also include the effect on ulcer index (UI) parameter that is based upon the area of the colon covered by ulceration relative to the total isolated area of the colon. The decrease in UI by CHME and CHnHE treatments at all doses as observed in the present study is due to a decrease in the extent of ulceration because of the phenolic and flavonoid contents of these extracts. A previous study that was performed to explore the activity of polyphenols in UC also reported similar findings (Salama et al., 2022).

Oxidative stress acts as a major partner to the immune system in the pathogenesis of UC and occurs due to the imbalance between the oxidant and antioxidant systems in the colon. This imbalance leads to increased lipid peroxidation (Alsharif et al., 2022) and decreased levels of antioxidant enzymes such as superoxide dismutase (SOD) in colon tissues. Decrease in SOD activity results in compromised detoxifying activity and ultimately free radicals create an oxidative and nitrosative storm (Elmaksoud et al., 2021). Restoration of SOD level in colon tissues by CHME and CH*n*HE improved the antioxidant capacity of colon tissues and this effect may be due to the presence of syringic acid in both extracts as this phytochemical compound has been reported to increase SOD activity in a previous study (Cao et al., 2016). Lipid peroxidation increases the production of MDA via induction of lipid peroxidation in the colon wall and the involvement of MDA in UC is well established. By inducing mitochondrial damage, MDA increases colon wall permeability and apoptosis with resultant ulceration and bleeding (Mierzwa et al., 2022). Excessive production of nitric oxide in ulcerative colitis as observed in CCG is due to increased activation of inducible nitric oxide synthase (iNOS) that is activated by pro-inflammatory cytokines such as TNF-α. Nitric oxide contributes in the pathogenesis of UC by promoting further generation of free radicals and infiltration of neutrophils and macrophages. The resultant pathological features including edema, erythema, inflammation, and diarrhea occur in correlation with nitric oxide levels in serum and colon tissues (Kamalian et al., 2020). A decrease in tissue level of MDA and nitric oxide with CHME and CH*n*HE treatments indicated a decrease in corresponding pathogenic features. The ability of these plant extracts to reduce MDA and NO can be attributed to the presence of tannins, sterols, and alkaloids as reported by a similar study (Adjouzem et al., 2020). Furthermore, CHME and CH*n*HE contain phenolic acids and flavonoids as well which also synergistically contribute to reducing oxidative stress in UC and this finding is in accordance with a previous study about these polyphenols (Keyvanara et al., 2023).

COX-2 enzyme is a critical member of an interconnected and complex network of biochemical mediators involved in the pathogenesis of UC. It regulates the production of pro-inflammatory cytokines and apoptotic mediators via the production of PGE2 and even is involved in malignant complications of UC. Its level in the inflamed colon is increased as was observed in the present study (Kjærgaard et al., 2020). CHME and CH*n*HE normalized the COX-2 level exhibiting the anti-inflammatory potential that may be attributed to polyphenols detected in both extracts as also observed in another study (Abdallah et al., 2020).

Histopathological alterations in UC involve upregulation of pro-inflammatory cytokines and downregulation of anti-inflammatory cytokines (Xue et al., 2023). NF-κB is one of the principal pro-inflammatory mediators that leads the inflammation in UC by promoting infiltration of neutrophils and macrophages in the colon wall (Laurindo et al., 2023). NF-κB via IL-1β also upregulates the expression of COX-2 enzyme that already has well-established involvement in inflammation (Teng et al., 2021). A dose-dependent decrease in inflammation score exhibited by CHME and CH*n*HE can be attributed to caffeic acid identified in both extracts that have the potential to block COX-2 via inhibition of NF-κB as reported by a similar study (Zielińska et al., 2021). Infiltrated neutrophils and macrophages downregulate the expression of anti-inflammatory cytokines such as IL-4 and IL-10 (Chen et al., 2021). Increased expression of IL-4 and IL-10 is needed for amelioration of the pathological architecture of UC. An increase in these anti-inflammatory cytokines by CHME and CH*n*HE can be attributed to their gallic acid constituent as also reported by another study (Zhu et al., 2019). Loss of goblet cells as observed in CCG has been linked with oxidative stress mediators such as MDA that have apoptotic potential (Elhassan, 2022). CHME and CH*n*HE prevented the loss of goblet cells perhaps due to the presence of quercetin which being a flavonoid opposes the oxidative stress markers such as MDA and this finding is in accordance with a previous work (Hu et al., 2022). Colon wall thickening as exhibited in CCG is due to the infiltration of polymorphonuclear cells in the colon wall (Qian et al., 2020). Both extracts of *C. haematocephala* in the current study exhibited restoration of colon wall thickness owing to the presence of sinapic acid in CHME and CH*n*HE. Sinapic acid has been found to block the infiltration of polymorphonuclear cells in colon wall (Qian et al., 2020).

Due to the nonspecific binding of plasma conjugates, quantification of cytokine proteins by ELISA has less certainty and the level of cytokine proteins in plasma is lower than that of their corresponding mRNA (Li et al., 2022). Furthermore, ELISA cannot be used to measure the cytokines stored intracellularly. Hence, cytokines do not retain stability in plasma owing to these concerns (Israelsson et al., 2020). In contrast to cytokine proteins, cytokine mRNA is more stable and is the principal regulator of cytokine synthesis (Mino et al., 2013). Due to these uncertainties associated with cytokine protein analysis by ELISA, cytokine quantification was done in the present study by measuring cytokine mRNA using RT-qPCR which is a widely used tool for cytokine expression profiling (Cerda et al., 2020). As stated earlier, pathogenesis of ulcerative colitis involves the upregulation of pro-inflammatory and downregulation of anti-inflammatory cytokines. Restoration of these cytokines as exhibited by CHME and CH*n*HE treatments in present study is also required for disease remission. NF-κB is among the major regulators of pathogenesis in UC as it promotes the expression of TNF-α and also activates COX-2 (Feng et al., 2022). Treatment with CHME and CH*n*HE decreased the mRNA expression of NF-κB and TNF-α probably due to the presence of phenolic acids as this assumption was also proposed by a previous work (Kim et al., 2021). Downregulation of anti-inflammatory cytokines such as IL-4 and IL-10, as observed in CCG, participates in UC because anti-inflammatory cytokines decrease the expression of pro-inflammatory cytokines such as TNF-α. Upregulation of IL-4 and IL-10 by all doses of CHME and CH*n*HE may be attributed to the presence of flavonoids as also documented by another study on flavonoids (Shukla et al., 2019).

Prevention of macroscopic damage, detoxification of oxidative stress, and amelioration of histopathological abnormalities as exhibited by all doses of CHME and CH*n*HE in comparison to prednisone are based upon their anti-inflammatory and anti-oxidant actions. Highest effects were whown by 750 mg/kg doses of CHME and CH*n*HE in all paraeters. These actions of CHME and CH*n*HE can be proposed as the basis of the curative potential of *C. haematocephala*in similar to a previous study on different plant extracts which documented that the anti-inflammatory actions of phytochemicals are mandatory for curating the UC (Zhou et al., 2021). Though, CHME and CH*n*HE have exhibited comparable anti-ulcerative colitis activities by restoring almost all pathological derangements of UC. However, CH*n*HE has possessed higher curative potential, as compared to CHME probably because of its greater phenolic and flavonoid contents as compared to CHME. Furthermore, the phytochemicals contained in CH*n*HE are lipophilic and hence may have somewhat higher colon tissue distribution than water-soluble ingredients of CHME. Further that prednisone has shown significantly favorable effects on inflammatory markers in acetic acid-induced UC comparable to CHME and CH*n*HE, but the effects of prednisone on macroscopic damage and histopathological scores were significantly less as compared to 750 mg/kg doses of CHME and CH*n*HE. So, just exhibiting the effect on cytokines cannot confer the potential to cure UC. Restoration of all pathological features of UC is needed for possessing curative potential in UC as CHME and CH*n*HE have exhibited in the current study.

## 5 Conclusion

Based on the findings of this study, it can be concluded that *Calliandra haematocephala* has significantly ameliorated the pathogenic features of UC. Methanolic and *n*-Hexane extracts of *Calliandra haematocephala* exhibited anti-ulcerative colitis activity by decreasing macroscopic damage, oxidative stress, and pathological score. Upregulation of anti-inflammatory cytokines (IL-4 and IL-10), downregulation of pro-inflammatory cytokines (TNF-α and NF-κB), decreased COX-2 level, detoxification of NO and MDA, and increased level of SOD can be proposed as underlying mechanisms of anti-ulcerative colitis activity of CHME and CH*n*HE. All these mechanisms can be attributed to the presence of phytochemicals in these extracts and *n*-Hexane exhibited greater curative potential perhaps because of its higher phenolic and flavonoid contents than that of methanolic extract. Hence, owing to the outcomes of the present study, *Calliandra haematocephala* can be considered as a potential cure for UC after bioassay-directed isolation of bioactive phytochemicals and clinical evaluation.

## Data Availability

The raw data supporting the conclusion of this article will be made available by the authors, without undue reservation.
